# Observational Study of Antibiotic Usage at the Children’s Clinical University Hospital in Riga, Latvia

**DOI:** 10.3390/medicina54050074

**Published:** 2018-10-23

**Authors:** Inese Sviestina, Dzintars Mozgis

**Affiliations:** 1 Faculty of Medicine, University of Latvia, Raiņa bulv., LV-1586 Riga, Latvia; 2 Children’s Clinical University Hospital, Vienibas avenue 45, LV-1004 Riga, Latvia; 3 Public Health and Epidemiology Department, Riga Stradiņš University, Dzirciema street 16, LV-1007 Riga, Latvia; dzintars@mozgis.lv

**Keywords:** defined daily dose, hospitalized children, parenteral antibiotics, point prevalence survey, third-generation cephalosporins

## Abstract

*Background and objectives:* Many pediatric patients have been treated with antibiotics during their hospitalization. There is a need to improve antibiotic prescribing for pediatric patients because many of these prescriptions are inappropriate. Antibiotic consumption analysis was conducted at the Children’s Clinical University Hospital to identify targets for quality improvement. *Materials and Methods:* A one day cross-sectional point prevalence survey (PPS) was conducted in May and November 2011–2013 using a previously validated and standardized method. The survey included all inpatient pediatric and neonatal beds and identified all children receiving an antibiotic treatment on the day of survey. Total consumption of systemic antibiotics belonging to the ATC J01 class (except amphenicols (J01B) and a combination of antibacterials (J01R)) was also analyzed by using a defined daily dose (DDD) approach and antibiotic drug utilization (90%DU) for the period 2006–2015. Results were compared with results in 2017 using the DDD and 90%DU methodology. *Results:* The most commonly used antibiotic group in all PPS, except in May and November 2011, was *other* β-lactam antibiotics (J01D): 42 (40%) prescriptions in May 2013 and 66 (42%) and November 2011. In 2006–2015 and also in 2017, the most commonly used antibiotic groups were penicillins (J01C) and *other* β-lactam antibiotics (J01D)—76% (90%DU) of the total antibiotic consumption registered in 2006, 73% in 2015 and 70% in 2017. Starting in 2008, amoxicillin was the most commonly used antibiotic at the hospital. The usage of ceftriaxone increased from 3% in 2006 to 13% in 2015, but decreased in 2017 (7%). *Conclusions:* Study results from 2006–2015 showed that there was a need to establish a stronger antibiotic prescribing policy in the hospital reducing the use of broad-spectrum antibiotics (especially 3rd generation cephalosporins) and increasing the use of narrower spectrum antibiotics. It was partly achieved in 2017 with some reduction in ceftriaxone use.

## 1. Introduction

Growing antimicrobial resistance has been recognized as a worldwide threat to public health [1,2]. Therefore new solutions are needed to improve antibiotic use. Gerber et al. found that up to 60% of antibiotics are still used incorrectly in hospitals (e.g., the use of broad-spectrum antibiotics instead of narrow-spectrum antibiotics, the administration of intravenous antibiotics instead of the use of or switching to oral antibiotics) [3]. According to some studies (Smith et al. and Dellit et al.) the majority of pediatric patients have been treated with antibiotics during their hospitalization and many of these prescriptions were inappropriate [4,5,6]. One of the latest World Health Organization reports on antibiotic resistance states that “there are significant gaps in surveillance, and a lack of standards for methodology, data sharing and coordination” [7]. The Ministry of Health of the Republic of Latvia founded the “Coordination Committee for Antimicrobial Resistance Limitation”, with the aim to introduce a national plan on antibiotic resistance, as well as the correct and rational use of antibiotics in Latvia. Although this plan is already written, it has still not been approved by the Ministry, perhaps due to higher priorities. It is impossible to introduce the correct and rational use of antibiotics without identification of the current position with regard to antibiotic consumption.

Although there are many reviews on interventions as to how to improve antibiotic prescribing practices, e.g., Davey et al. reports [8] the limited amount of reliable data available on antibiotic use in children in hospitals compared with adult data, although antibiotics are amongst the most frequently prescribed medicines administered to children [9,10]. The American Academy of Pediatrics guidelines [5] state that only a few studies have focused on hospitalized newborns, children, and adolescents. The study performed in 40 pediatric hospitals across the United States of America established that during their hospital stay up to 60% of children received at least one antibiotic [11]. The study concluded that children at some hospitals were undertreated with antibiotics and thus could be exposed to the risk of mistreatment and that some hospitalized children have received excessive antibiotic therapy and thus were unnecessarily exposed to the risk of developing antibiotic-resistant infections. Godbout et al. found that although antimicrobial stewardship programs (ASP) had been formally recommended in the US since 2007 by the Infectious Diseases Society of America, there were still very few studies evaluating the effectiveness of pediatric stewardship programs. They focused on studies done in the US with implemented interventions and completed assessments of interventions. They found only 17 studies which met the inpatients inclusion criteria [12]. Brett et al. focused on ASPs in Europe and they found that these programs were not widely used in pediatrics. For instance, they did not find any publications on ASP studies involving multiple interventions and activities [13]. It was only in 2012 when Europe had its first European-wide point prevalence survey (PPS) on antibiotic use in hospitalized pediatric patients [14]. Results of this study showed significantly lower antibiotic prevalence rates in the Northern European region comparing with the Southern European hospitals. Authors also found that there were significantly higher proportions of antibiotic use in hematology/oncology wards and pediatric intensive care units when compared with the overall prevalence in pediatric wards. Another conclusion was that comprehensive antibiotic guideline recommendations were generally lacking from European pediatric hospitals and that there were multiple antibiotics and combinations used for most infections [15]. A Children’s Clinical University Hospital (CCUH) together with pediatric and neonatal wards from eight other Latvian hospitals participated in the “Antibiotic Resistance and Prescribing in European Children” (ARPEC) study but these results were difficult to compare because the CCUH results comprised the largest component of all the results. The CCUH also participated in the European Centre for Disease Prevention and Control (EDCD) PPSs in 2012 [16] and 2016. The ECDC PPS methodology differs from the ARPEC protocol therefore the ECDC PPSs results were not included in our study. Some of the pediatric and neonatal wards from other Latvian hospitals also participated in these ECDC PPSs but these results were not separately analyzed from adult data.

Although the CCUH has participated in the ARPEC and EDCD PPSs studies, a systematic antibiotic consumption analysis was not conducted at the CCUH. Therefore, there was a lack of comprehensive information on antibiotic usage trends at the hospital.

The objectives of this study were to analyze the antibiotic consumption trends in the CCUH in order to obtain information on antibiotic usage as well as to analyze the consumption trends after introduction of some ASPs into the hospital.

We hypothesized that there was a high use of *other* β-lactam antibiotics (J01D) and especially ceftriaxone in the hospital that could be reduced using ASP.

## 2. Materials and Methods

### 2.1. Study Design

Two methods were used: First, the repeated PPSs during the period from 1 January 2011 to 31 December 2013 twice a year (in May and November each year). In total there were six PPS conducted. Pediatric surgical wards were not audited on a Monday in order to capture information about prophylaxis in the previous 24 h (duration of prophylaxis was either 1 dose, or several doses during one day, or >1 day). Wards were not audited on holidays or on weekend days. Only systemic antibiotics belonging to the ATC J01 group were included in the study. These antibiotic groups were: Tetracyclines (doxycycline) (J01A), β-lactam antibiotics, penicillins (J01C), *other* β-lactam antibiotics (J01D), sulfonamides and trimethoprim (J01E), macrolides and lincosamides (J01F), aminoglycosides (J01G), quinolones (J01M), and other antibiotics (J01X). We did not have amphenicols (J01B) or combinations of antibacterials (J01R) in the hospital. The PPS protocol developed and validated by ARPEC was used in this study and full details of the ARPEC methodology were described elsewhere by Versporten et al. [14].

Second, following the ATC/DDD guideline [17,18] the defined daily dose (DDD) method and antibiotic drug utilization (90%DU) method [19] were used for the period 2006–2015 and these results were compared with 2017 results. Consumption rates were expressed as DDD, DDD/100 bed days (BD) and DDD/100 inpatients.

### 2.2. Setting

This study was conducted at the CCUH, a 300 beds tertiary-care hospital in Riga, Latvia. This is the only children’s hospital in the country with all medical specialties available except for organ transplantation. There were two independent pediatric hospitals (both in Riga) until 2005. These hospitals merged with each other during 2005. Therefore we decided to analyze hospital data starting in 2006 when the merging process was finished.

### 2.3. Participants

All inpatients under 18 years old present on the ward at 8:00 am on the day of the PPSs were included in the PPSs. Detailed data were recorded only for patients with active antimicrobial prescriptions at 8 am on the day of the survey. Following to the ARPEC protocol [14] day surgery, day hospital admissions, emergency admissions after midnight, patients on psychiatric wards and children younger than 18 years admitted on an adult ward were excluded.

### 2.4. Data Collection

The hospital has an established ward-based clinical pharmacist service and a clinical pharmacist collected all of the data. The following data were collected for the PPS: Patient demographic details, e.g., gender, age, weight (birth weight and gestational ages for neonates), prescribed systemic antimicrobials (ATC J01C), indication, single dose and number of doses per day, frequency, route of administration, reason for treatment, prophylaxis (e.g., medical or surgical).

Antibiotic consumption data were obtained from the hospital pharmacy electronic database. The amount of antibiotics distributed from the pharmacy to the wards was taken into account. The total amount of every antibiotic used per year was converted in terms of grams. The number of treated patients and the number of hospital bed-days were used to characterize intensity of patients’ treatment. In addition, the analysis considered all data related the average duration of treatment. Information on bed-days, number of treated patients and average duration of the said treatment were obtained from the CCUH eHealth and Statistics Department. The day of the patient’s hospitalization as well as discharge was considered as one day. 

Total consumption of antibiotics at the CCUH was also analyzed by using the 90%DU method. Antibiotics were ranked by amount of DDD. Antibiotics, which accounted for 90% of the total volume of use, were specifically noted [19].

### 2.5. Intervention

Several ASPs were introduced, e.g., antibiotic surgical prophylaxis recommendations, antibiotic order (paper) forms, electronic prescribing, and yearly antibiotic consumption analysis in different clinics (with a special focus on the Surgery clinic). Therefore results from 2016 were not included in the study.

### 2.6. Main Outcome Measure

Antibiotic usage analysis was done by using PPS and DDD methodology in order to get information about existing situation in the hospital that was compared before and after the ASP interventions using the DDD methodology.

### 2.7. Data Analysis

This was a descriptive analysis. Two different metrics of antibiotic prescribing were compared: (1) the proportion of children on antibiotics (prevalence rate) with 95% CIs; (2) the DDD per 100 bed-days (DDD/100 BD) and DDD per 100 inpatients (DDD/100 inpatients). The denominator “inpatients” was defined as the sum of inpatients in the hospital in a particular year. The denominator “bed-days” was defined as the sum of bed-days in the hospital (except those wards that were not analyzed) in a particular year. We analyzed both patients on antibiotics and numbers of antibiotic prescriptions because many patients received more than one antibiotic during PPS.

### 2.8. Quality Indicators

We explored two different inpatient antibiotic prescribing quality indicators: (1) the total amount of antibiotics used using both metrics, the proportion of children receiving antibiotics in different age bands and DDD/100 BD and DDD/100 inpatients as well as 90% DU. (2) The use of ceftriaxone and the use of penicillins (ampicillin, amoxicillin and penicillin G) were analyzed because the correct use of ceftriaxone is essential in order not to increase resistance and penicillins still could be used in many situations instead of the 3rd generation cephalosporins and especially ceftriaxone. The proportion of prescribed antibiotics during PPS and as well as the amount of these antibiotics used in DDD/100 BD, DDD/100 inpatients and 90% DU were analyzed. 

The statistical analysis was performed by using IBM SPSS Statistics Version 20.0 statistical software package (IBM SPSS Statistics Version 20, SPSS Inc., Chicago, IL, USA) program and Microsoft Excel program. Patients’ data were analyzed by using descriptive statistical methods (percentage proportion). Nominal data were described as the quantity (n) and percentage with 95% confidence interval (CI). Categorical data were analyzed with chi-square test (2 × 2 tables). Categorical (qualitative) data were also described as the quantity and percentage proportion. A Pearson’s correlation was used to examine the relation between two variables. Data with *p*-value < 0.05 were regarded as statistically significant. 

### 2.9. Ethics

The study was conducted in accordance with the Declaration of Helsinki, and the protocol was approved by the Ethics Committee of the Riga Stradiņš University (Project identification code 06102011).

## 3. Results

### 3.1. Point Prevalence Surveys’ Results

There were between 26% and 38% of patients on antibiotics during the PPSs with a highest number of patients receiving antibiotics in the November PPSs. The proportion of children on antibiotics was higher amongst children aged 1–5 years except for the PPS carried out in November 2013. The biggest number of patients received one antibiotic, although there were patients who received up to 5 antibiotics at the same time (Table 1). These were mostly patients on the hematology/oncology ward.

There was a statistically significant difference in males and females receiving antibiotics only in May 2012 (chi-square test 5.3059, *p* = 0.02).

The most commonly used antibiotic group in all of the PPSs, except in May and November 2011, was *other* β-lactam antibiotic (J01D). Ceftriaxone was the fifth most frequently used antibiotic in May 2011: 12 (9%; CI 3.9–13.1) prescriptions, but in November 2013 it became the most often used antibiotic: 23 (17%; CI 10.9–23.9) prescriptions. There were only intravenous and oral routes of administration and intravenous route was the main prescribed route (76–86% of all prescriptions) (Table 2).

**Figure 1:**
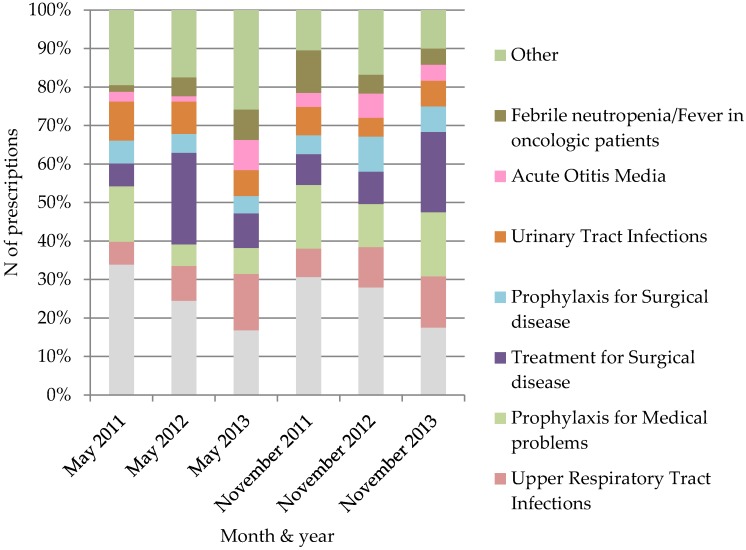
Reason of antibiotic use in PPSs during the period of 2011–2013.

The most common reason for antibiotic usage in pediatric patients in all of the PPSs (except November 2013) was for lower respiratory tract infections (LRTI). A diagnosis of LRTI was recorded in 15 (17%) prescriptions in May 2013 and 40 (34%) prescription (the highest percentage of prescriptions) in May 2011 (Figure 1).

The Hospital microbiology laboratory data showed that, for instance, 19% (of 335 tested cultures) of coagulase negative Staphylococcus were sensitive to penicillin, 81% (92) of *Enterococcus* sp., 14% (7) of *E. faecium* and 83% (47) of *E. faecalis*. 98% (354) of coagulase negative Staphylococcus were sensitive to oxacillin. Sensitivity to ampicillin was 91% (97) of *Enterococcus* sp., 52% (537) of *E. coli*. Sensitivity to cefotaxime was 92% (566) of *E. coli* and 85% (67) *K. pneumoniae*.

### 3.2. Defined Daily Dosage Method’s Results

The number of treated patients decreased from 26,055 patients in 2006 to 15,445 patients in 2015 (14,530 patients in 2017), and the number of bed-days decreased from 149,125 in 2006 to 101,057 bed-days in 2015 (70,894 bed-days in 2017). The average duration of treatment decreased from 5.7 days in 2006 to 5.1 days in 2015 (4.9 days in 2017). 

In total there were 91 antibiotic formulations used during the period of 2006–2015: 44 (48%) intravenous and 47 (52%) oral formulations. There were 33 (49%) intravenous formulations and 34 (51%) oral formulations in 2017.

The total antibiotic consumption in DDD/100 BD increased by 25% during the study period. Antibiotic consumption (DDD/100 inpatients) slightly increased: From 226 DDD/100 inpatients in 2006 to 253 DDD/100 inpatients in 2015.

#### Antibiotic Groups Used during the Study Period

During the period of 2006–2015 the most commonly used antibiotic groups were β-lactam antibiotics, penicillins (J01C) and other β-lactam antibiotics (J01D), which when combined accounted for 76% of the total antibiotic consumption (DDD) registered in 2006 and 71% in 2015. A similar trend was also observed in DDD/100 BD (Table 3). Statistically the 1st generation cephalosporin consumption decreased significantly in respect of all three indicators (e.g., DDD/100 BD r = −0.82, *p* < 0.05), but the 2nd generation, and especially 3rd generation cephalosporin consumption, statistically significantly increased (e.g., DDD/100 BD r = 0.90 and r = 0.92, *p* < 0.05). Ceftriaxone consumption increased in respect to all three indicators: DDD, DDD/100 BD and DDD/100 patients. The consumption of all other antibiotic groups (tetracyclines (J01A), sulfonamides and trimethoprim (J01E), macrolides, lincosamides and streptogramins (J01F), aminoglycoside antibacterials (J01G), quinolone antibacterials (J01M) and *other* antibacterials (J01X)) had not a statistically significant growth or reduction. In 2017 β-lactam antibacterials, penicillins (J01C) antibiotic group usage increased and *other* β-lactam antibacterials (J01D) group antibiotic use slightly increased (Table 3).

Comparing results of years 2017 and 2015 ceftriaxone usage has significantly decreased (Table 4).

In the Surgery clinic ceftriaxone consumption increased from 1.9 DDD/100 BD in 2006 to 5.5 DDD/100 BD in 2013 and 21.3 DDD/100 BD in 2015. In 2017 consumption decreased to 5.7 DDD/100 BD. Ampicilling consumption decreased from 12 DDD/100 BD in 2006 to 7.6 DDD/100 BD in 2013 and increased to 16.8 DDD/100 BD in 2015 and 21.7 DDD/100 BD in 2017. But cefazolin consumption increased from 2.5 DDD/100 BD in 2006 to 3.7 DDD/100 BD in 2013, 8.4 DDD/100 BD in 2015 and 9.7 DDD/100 BD in 2017. 

### 3.3. Antibiotic Consumption 90%DU Analysis

**Figure 2:**
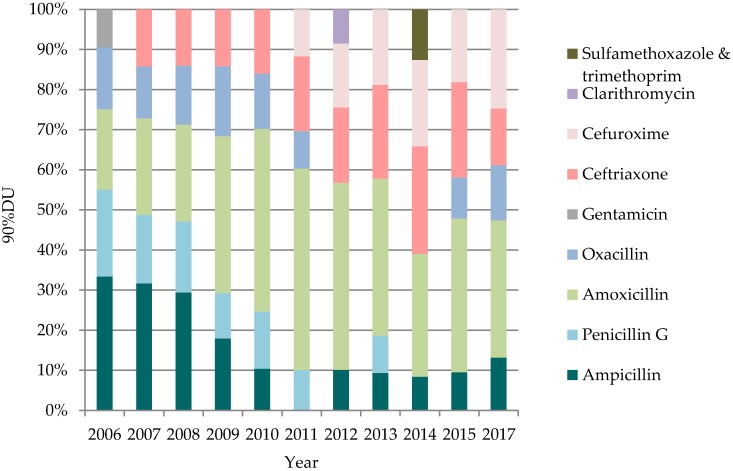
Five most commonly used AB during the period of 2006–2015 and 2017 (90%DU).

During the period of 2006–2015 the total number of antibiotics prescribed ranged from 36 antibiotics (in 2006) to 30 antibiotics (in 2012, the lowest number) and 36 antibiotics in 2017. Ninety percent of all used antibiotics equaled 14 antibiotics in average. In 2006–2015 and also in 2017 the most commonly used antibiotic groups were β-lactam antibiotics, penicillins (J01C) and other β-lactam antibiotics (J01D)—76% (90%DU) of the total antibiotic consumption registered in 2006, 73% in 2015 and 70% in 2017. Starting from 2008, amoxicillin (J01CA04) was the most commonly used antibiotic at the hospital. During the studied period, it was also the only antibiotic that was among five most frequently used antibiotics (Figure 2). Amoxicillin consumption increased from 12% in 2006 to 21% in 2015 but decreased in 2017 (18%). Although ampicillin (J01CA01) was among 90%DU antibiotics, during the studied period the ampicillin usage decreased significantly: From 21% of the total consumption in 2006 to 5% in 2015 and slightly increased in 2017 (7%). The usage of ceftriaxone (J01DD04) increased from 3% in 2006 to 13% in 2015, but its usage significantly decreased in 2017 (7%). Starting 2010–2015 ceftriaxone was the second most commonly used antibiotic. 2017 results showed that four penicillin group antibiotics were among the top five antibiotics prescribed (Figure 2).

## 4. Discussion

In 2003, 2005, 2007 and 2011 there several national level PPSs were conducted in Latvia [20]. Nevertheless, the usage of antibiotics in hospitalized pediatric patients as a separate patient cohort has been analyzed only since 2011. Pediatric patients are quite often analyzed together with adults [21,22] or there are different nuances in PPS protocols, e.g., information about antibiotic doses are not collected [23,24].

The PPSs results showed that there was a high use of the 3rd generation cephalosporins, especially ceftriaxone consumption in the CCUH in general. More and more cephalosporin and quinolone use has been linked to development of resistance. These are broad-spectrum antibiotics, which reach high concentrations in the body and are excreted over a relatively long time. The most appropriate antibiotic is that of the narrow-spectrum penicillin group. PPS methodology does not provide an explanation in particular for the high use of the 3rd cephalosporins that made a biggest part of other β-lactam antibiotics group (J01D) consumption increases in the hospital (the tendency what we found by using DDD methodology). The PPSs only confirms that the 3rd generation cephalosporins were among the most often used antibiotic group in different fixed time periods of the year when the PPSs were done.

Laine et al. [25] found a reduction in the consumption in penicillin G in their hospital in 2011–2012 which took place at the same time pneumococcal conjugate vaccine was introduced to the national immunization program in Finland (at 2010). We also had a reduction in the consumption of penicillin G at the same time. We do not have data of the numbers of invasive pneumococcal infections, therefore we can only assume that it could be one of reasons for penicillin G reduction. The increase of ceftriaxone usage could be partly explained by the use of ceftriaxone in surgical prophylaxis and because the administration takes less time (being administered only once or twice a day compared with cefazolin or amoxicillin) and would need less personnel resources. There could also be some cultural differences that influenced the ceftriaxone usage. One of the children’s hospitals, before merging, mostly used cefotaxime (selecting between 3rd generation cephalosporins) while another hospital, the largest one used ceftriaxone. But approval or disapproval of this thesis needs a different study, e.g., some qualitative research. Our specialists still have a free choice regarding which guidelines to use. Their choice is often based on American or British guidelines, although the resistance situation is different in these countries. Another explanation could be that as a tertiary care hospital, it had patients who have already received antibiotics prior to hospitalization in primary care or in another hospital before they were transported to CCUH. If the empirical treatment has started with sulfamethoxazole/trimethoprim or 2nd generation cephalosporins, it is not always possible to switch to β-lactam antibiotics, penicillins (J01C) or the narrow spectrum penicillins. The reduction of ceftriaxone use might be partly explained by the ASP activities performed in the Surgery clinic.

Several interventions were done to decrease unnecessary and incorrect use of antibiotics, e.g., recommendations (for surgical prophylaxis, appendicitis treatment, gastroenteritis treatment etc.) and a special antibiotic prescription form were introduced in the hospital. An antibiotic stewardship team was also established in the hospital. The special focus was on the surgery clinic to reduce the use of ceftriaxone both for prophylaxis and treatment. There were regular clinical pharmacist visits and also visits by the chief physician to the surgery clinic ward rounds. Ceftriaxone was removed from the surgical prophylaxis protocol and if used, then it was recommended to reduce the duration of treatment whenever possible.

The route of antibiotic administration is also one of the quality indicators of antibiotics usage. There was a high number of parenterally administrated antibiotics at CCUH. The switch from parenteral to oral antibiotics depends upon the preference of each physician—the hospital makes only general recommendations. The usage of parenteral antibiotics also indicates that the necessity of antibiotics usage was not always evaluated after the first 48–72 h [26]. This warning was introduced into the electronic prescription system from October 2018. The CCUH results are similar to those obtained from other Latvian hospitals where 3rd generation cephalosporins were among the most frequently used antibiotics (previously these were the 1st generation cephalosporins) as well as the parenteral route of administration being the most commonly used route [20]. The CCUH results cannot be generalized because there is only one children’s hospital in the country therefore all seriously ill patients are transferred to CCUH. We can try to compare our results only with published data. The problem is that even if other authors [3,4,5,6,26] use the same methodology, e.g., DDD, PPS, results are not always presented in the same way, which makes comparison difficult. Comparison is possible only if we have been a part of some bigger study, e.g., Versporten et al. [14].

### Strength and Limitations

By means of the PPSs, necessary information was obtained from the patients’ medical documents. Therefore there was less possibility of collecting wrong data as compared with the DDD approach where data were obtained from the pharmacy and wards. In addition, unlike the DDD method, where aggregated data (hospital in general or particular ward) were used, in PPSs individual (patient specific) data were used. The PPS methodology was useful for our study, because it enabled obtaining information on antibiotics usage for a specific time period as well as on antibiotics usage tendencies (because the PPSs were repeated). This method also allowed us to obtain information on spectrum of used antibiotics in the hospital in general and on specific wards in particular. Results of the PPSs showed similar antibiotic usage tendencies as compared to the DDD results. The strength of the DDD method was that it was possible to obtain information about the antibiotic consumption (DDD) and the intensity of antibiotic usage (DDD/100 BD and DDD/100 inpatients) in the hospital in general and on the hospital ward or clinic in particular. Both methods were useful for our study because the DDD method, as with the PPS method, did not require substantial financial resources to perform the analysis and both protocols were not very complicated. All of this was important because CCUH did not have extra financial and human resources to carry out the antibiotic consumption analysis. This study was be done by the clinical pharmacist in collaboration with both physicians and nurses when necessary.

The PPS method also had some limitations, e.g., analysis was performed only on the patients who received antibiotics at a specific point in time, but it was impossible to obtain information for patients, who ought to have received antibiotics but for some reason did not actually receive them. The PPS method did not provide information on the total antibiotics consumption over a longer period, as it recorded the situation at a particular point in time. In general, cross-sectional studies, that also include the PPS, do not allow analysis of the incidence (e.g., time when the antibiotic therapy is started). One of important limitations of the DDD method was that it did not show the real antibiotic consumption. This is because DDDs is an artificially created unit of measurement and does not necessarily reflect the recommended or prescribed daily dose [27,28]. Antibiotic consumption analysis in hospital wards was complicated by a situation where the structure of wards changed significantly during 2011–2013 and some changes continued until 2017: Some wards were merged together, some wards changed medical profiles, and some wards were closed while the hospital pharmacy continued to count antibiotics by using the same old administrative ward principle and did not take into account the new medical profiles. Therefore there could be a situation that on the ward where previously patients with gastroenterology and endocrinology issues were hospitalized, after the restructuring also had patients with other conditions, such as rheumatology and nephrology problems. But the pharmacy still regarded it as a ward as defined previously. These administrative changes had a negative influence on the quality of the results and antibiotic consumption analysis on the wards had no significant value. Therefore we did not analyze the situation on specific wards by using the DDD methodology. Although DDDs has been one of the most common units used in measuring antibiotic use in children, the WHO DDD methodology is not applicable in children due to the vast differences in body weight within this particular population. This was one of the most important limitations in the context of antibiotic consumption studies in children in our hospital. Unfortunately, there is no consensus regarding the optimal metric to assess pediatric antibiotic usage, which is an important limitation in pediatric patients. Such methods as recommended or prescribed daily dose, days of therapy (DOT) or length of therapy (LOT) also have limitations. The DDD method (together with use of denominators “bed-days” and “inpatients”) could be used only to monitor changes over time to alert prescribers that there could be problems with some antibiotics being prescribed or administered incorrectly. This method is still used in pediatric studies [17,19,29,30]. Another important limitation that did not allow us to choose a different method of analysis was the situation that all patients’ medical information was recorded on paper and it would have taken a lot of time and human resources to evaluate the antibiotic consumption tendencies in the hospital based on individual patients’ medical records. Therefore, we decided to use the DDD methodology with all of its limitations.

There are no established similar principles for antibiotic consumption analysis in Latvian hospitals, and almost all consumption studies are voluntary and based on researcher enthusiasm. Nevertheless, this is also a problem in many other countries [31,32]. At the moment hospitals in Latvia can choose to conduct or not to conduct antibiotic consumption studies, as well as what methodologies to use. It was possible to use the DDD method in this study, because one of the tasks was to analyze antibiotic consumption trends provided by this method. The growing antimicrobial shortages problem should also be taken into account at least in the future. It is already reported to be a problem in the USA [33]. In Europe, the European Association of hospital pharmacists conducted a survey (March–June 2018) on this very topic. Therefore effective collaboration is needed between physicians and pharmacists.

## 5. Conclusions

Study results from 2006–2015 showed that there was a need to establish a stronger antibiotic prescribing policy in hospitals, reducing the use of the broad-spectrum antibiotics (especially 3rd generation cephalosporins) and increasing the use of narrower spectrum antibiotics as well as the use of oral antibiotics. Some reduction of ceftriaxone usage was achieved in 2017. However, all hospitals still need to improve their antibiotic prescribing policies.

## Figures and Tables

**Table 1:** CCUH patients’ characteristics (2011–2013).

Year	2011	2012	2013	2011	2012	2013
Month	May	May	May	November	November	November
Patients	N (%) (95% CI)	N (%) (95% CI)	N (%) (95% CI)	N (%) (95% CI)	N (%) (95% CI)	N (%) (95% CI)
Total number	418	395	335	424	358	320
Male	230 (55) (50–60)	215 (54) (50–59)	178 (53) (48–58)	225 (53) (48–58)	196 (55) (50–60)	159 (50) (44–55)
Female	188 (45) (40–50)	180 (46) (41–51)	157 (47) (42–52)	199 (47) (42–52)	162 (45) (40–51)	161 (50) (45–56)
Patients on antibiotics	125 (30) (26–34)	128 (32) (28–37)	88 (26) (22–31)	159 (38) (33–42)	130 (36) (31–41)	111 (35) (30–40)
Gender:						
Male	63 (50) (42–59)	59 (46) (38–55)	47 (53) (43–64)	90 (57) (49–63)	74 (57) (48–65)	57 (51) (42–61)
Female	62 (50) (41–58)	69 (54) (45–63)	41 (47) (36–57)	69 (43) (36–51)	56 (43) (35–52)	54 (49) (39–58)
Age groups:						
0–<1 month	20 (16)	10 (8)	10 (11)	19 (12)	12 (9)	19 (17)
≥1 month–<1 year	14 (11)	27 (21)	13 (15)	24 (15)	21 (16)	20 (18)
≥1–<5 years	52 (42)	33 (26)	32 (36)	54 (34)	36 (28)	24 (22)
≥5–<12 years	27 (22)	29 (3)	14 (16)	33 (21)	35 (27)	27 (24)
≥12–<18 years	12 (10)	29 (23)	19 (20)	29 (19)	26 (20)	21 (19)
	How many antibiotics received one patient:					
1 antibiotic	108 (86)	102 (79)	69 (79)	141 (88)	102 (79)	95 (85)
2 antibiotics	17 (14)	23 (18)	15 (17)	8 (5)	23 (18)	12 (11)
3 antibiotics	0	2 (2)	3 (3)	6 (4)	2 (1)	2 (2)
4 antibiotics	0	1 (1)	1 (1)	4 (3)	3 (2)	1 (1)
5 antibiotics	0	0	0	0	0	1 (1)

**Table 2:** Antibiotic groups used during PPSs in 2011–2013 and the route of administration.

Year	2011	2012	2013	2011	2012	2013
Month	May	May	May	November	November	November
Antibiotics (prescriptions):	N (%)	N (%)	N (%)	N (%)	N (%)	N (%)
Total N of prescriptions	142	157	106	192	162	132
Aminoglycosides (J01G)	11 (8)	15 (10)	9 (9)	11 (6)	13 (8)	12 (9)
β-lactam AB, penicillins (J01C)	51 (35.9)	46 (29.3)	32 (30.2)	65 (34)	49 (30)	23 (17)
Macrolides and lincosamides (J01F)	8 (6)	6 (4)	6 (6)	12 (6.3)	12 (7.4)	9 (6.8)
Other β-lactam AB (J01D)	46 (32)	66 (42)	42 (40)	63 (33)	67 (41)	54 41)
Quinolones (J01M)	1 (1)	3 (2)	2 (2)	0	0	5 (4)
Other AB (J01X)	7 (5)	10 (6)	7 (7)	17 (9)	9 (6)	10 (8)
Sulfonamides and trimethoprim (J01E)	18 (13)	11 (7)	8 (8)	24 (13)	12 (7)	19 (14)
Six most often used antibiotics:						
Ampicillin	25 (18)	10 (6)	9 (8)	17 (9)	5 (3)	6 (5)
Ceftriaxone	12 (8)	13 (8)	16 (15)	19 (10)	25 (15)	23 (17)
Amoxicillin	11 (8)	19 (12)	8 (8)	22 (11)	21 913)	7 (5)
Cefuroxime	9 (6)	22 (14)	9 (8)	12 (6)	18 (11)	15 (14)
Sulfamethoxazole & trimethoprim	18 (13)	11 (7)	8 (8)	24 (13)	12 (7)	15 (14)
Penicillin G	14 (10)	11 (7)	11 (10)	19 (10)	17 (10)	8 (6)
Route of administration:						
Intravenous	108 (76)	135 (86)	91 (86)	146 (76)	131 (81)	100 (76)
Oral	34 (24)	22 (14)	15 (14)	46 (24)	31 (19)	32 (24)

**Table 3:** Antibiotic groups used during the period of 2006–2015 and 2017 (DDD/100 BD and DDD/100 inpatients).

Year	2006	2007	2008	2009	2010	2011	2012	2013	2014	2015	2017
DDD/100 BD:											
Aminoglycosides (J01G)	3.9	4.0	3.3	3.3	2.5	3.3	3.0	3.6	3.5	2.3	2.3
β-lactam AB, penicillins (J01C)	23.0	24.2	19.7	20.4	22.0	22.0	19.8	19.7	14.2	18.7	21.2
Macrolides and lincosamides (J01F)	1.3	2.1	1.2	1.0	1.4	1.9	3.4	3.5	2.6	8.1	4.9
Other β-lactam AB (J01D)	6.8	10.3	9.2	8.3	9.4	11.2	12.2	16.4	16.7	15.7	16.8
Quinolones (J01M)	0.7	1.3	0.5	0.3	0.5	1.8	0.6	0.9	1.9	1.4	1.0
Tetracyclines (J01A)	0.2	0.5	0.6	0.4	0.5	0.6	0.4	0.2	0.4	0.3	0.2
Other AB (J01X)	1.9	2.7	1.1	2.2	2.3	2.4	3.3	4.0	3.4	4.3	5.2
Sulfonamides and trimethoprim (J01E)	1.6	1.6	2.0	2.1	1.8	0.5	0.4	0.8	3.2	2.4	2.7
DDD/100 inpatients:											
Aminoglycosides (J01G)	22	22	19	18	18	16	13	15	18	12	11
β-lactam AB, penicillins (J01C)	131	136	112	107	111	90	88	82	74	57	104
Macrolides and lincosamides (J01F)	7	12	7	5	7	9	15	15	13	16	24
Other β-lactam AB (J01D)	39	58	52	43	47	53	54	69	85	83	82
Quinolones (J01M)	4	7	3	2	3	9	3	4	10	7	5
Tetracyclines (J01A)	1	3	3	2	2	3	2	1	2	0.3	1
Other AB (J01X)	11	15	6	12	12	11	15	17	18	22	25
Sulfonamides and trimethoprim (J01E)	9	9	11	11	9	2	2	3	17	12	13

**Table 4:** Amoxicillin, ampicillin, penicillin G and ceftriaxone consumption during the period of 2006–2015 and 2017.

Year	2006	2007	2008	2009	2010	2011	2012	2013	2014	2015	2017
DDD/100 BD:											
Amoxicillin	4.9	6.5	5.3	8.8	11.5	13.5	13.0	11.3	7.7	10.8	9.8
Ampicillin	8.1	8.6	6.4	4.0	2.6	2.4	2.7	2.7	2.1	2.7	3.7
Penicillin G	5.2	4.3	3.8	2.5	3.6	2.7	1.9	2.7	1.4	1.2	0.7
Ceftriaxone	1.3	3.9	3.0	3.2	3.9	5.0	5.1	6.7	7.4	6.7	4.0
DDD/100 inpatients:											
Amoxicillin	27.8	36.5	29.8	46.2	57.9	64.7	56.2	47.1	40.2	56.8	47.9
Ampicillin	46.0	48.0	36.3	21.2	13.1	11.7	12.1	11.3	11.1	14.0	17.9
Penicillin G	30.0	25.8	21.7	13.3	18.0	13.0	8.3	11.2	7.3	6.5	3.5
Ceftriaxone	7.4	21.8	17.3	16.7	19.5	24.1	22.7	28.1	38.2	35.1	19.3
